# Strategies for Mitigating Sexual Desire Discrepancy in Relationships

**DOI:** 10.1007/s10508-020-01640-y

**Published:** 2020-02-07

**Authors:** Laura M. Vowels, Kristen P. Mark

**Affiliations:** 1 Department of Psychology, University of Southampton, Shackleton (Building 44), 46 Chamberlain Road, Southampton, SO17 1PS UK; 2 Department of Kinesiology and Health Promotion, University of Kentucky, Lexington, KY USA

**Keywords:** Sexual desire, Desire discrepancy, Sexual satisfaction, Relationship satisfaction, Mixed methods

## Abstract

Sexual desire discrepancy, when one member of a couple experiences more or less sexual desire relative to their partner, is among the main reasons for couples to seek therapy. A great deal of prior research has examined the complexity of sexual desire and the role of sexual desire discrepancy in long-term relationships, but little research has specifically examined strategies used to mitigate sexual desire discrepancy when it arises. Thus, the purpose of the present mixed methods study was to identify the strategies that individuals in long-term relationships use during times of desire discrepancy and to address whether the use of specific strategies influenced sexual and relationship satisfaction and sexual desire. We collected data from 229 participants and our thematic content analysis produced 17 strategies, divided into five main groups (disengagement, communication, engagement in activity alone, engagement in other activity with partner, and have sex anyway). Specific strategies were associated with sexual and relationship satisfaction but not with sexual desire. Specifically, partnered strategies were associated with higher levels of sexual and relationship satisfaction compared to individual strategies. Additionally, participants who reported that their strategies were very helpful had higher levels of sexual and relationship satisfaction compared to participants who found them somewhat helpful followed by not at all helpful. These results have implications for clinicians, educators, and researchers and highlight the importance of using effective strategies to deal with desire discrepancy and communicating about them in relationships. The use of effective strategies can have implications for overall couple well-being.

## Introduction

Sexual desire is of great interest to researchers, clinicians, and educators, in part because sexual desire has been linked to both relational (Mark, 2012; Mark & Murray, 2012) and individual (Lee, Vanhoutte, Nazroo, & Pendleton, 2016) outcomes and difficulties with sexual desire and desire discrepancy are among the most common reasons for couples to seek therapy (Ellison, 2002). Sexual desire can be conceptualized as a feeling of wanting to engage in a sexual activity (Basson, 2002), and desire discrepancy occurs when one partner’s desire is higher or lower than his or her partner’s (Mark & Murray, 2012). Several researchers have studied the maintenance of sexual desire and desire discrepancy in relationships (Davies, Katz, & Jackson, 1999; Mark, 2012; Mark & Lasslo, 2018; Santtila et al., 2007; Sims & Meana, 2010; Willoughby & Vitas, 2012). However, relatively little attention has been paid to the strategies people in long-term relationships use when only one partner is interested in sexual activity. Thus, the purpose of the present mixed methods study was to identify strategies that people use on days when their desire is out of sync with the partner and to see whether the use of specific types of strategies is associated with participants’ relationship and sexual satisfaction, and sexual desire.

A recent systematic review from Mark and Lasslo (2018) provides an overview and a conceptual model highlighting the myriad of factors that influence sexual desire and discusses ways to maintain sexual desire in relationships. Factors influencing sexual desire can be individual, interpersonal, and societal. Individual factors include attraction to one’s partner (Basson, 2000), hormones (Caruso et al., 2014; Mark, Leistner, & Garcia, 2016; Mass, Holldorfer, Moll, Bauer, & Wolf, 2009), stress (Ferreira, Narciso, Novo, & Pereira, 2014), and self-esteem (McCarthy & Wald, 2015; Murray & Milhausen, 2012a). Interpersonal factors include relationship length (Ainsworth & Baumeister, 2012; Klusmann, 2002; Murray & Milhausen, 2012b), satisfaction (Brezsnyak & Whisman, 2004; Ferreira et al., 2014; Shrier & Blood, 2016), communication (Ferreira et al., 2014; Murray & Milhausen, 2012a; Murray, Milhausen, & Sutherland, 2014), and emotional intimacy (Shrier & Blood, 2016). Societal factors include gendered expectations (Murray, 2018), egalitarianism (Brezsnyak & Whisman, 2004), and attitudes toward sex (Carvalho & Nobre, 2011). Some suggested mechanisms whereby couples can maintain sexual desire for each other include engaging in self-expanding activities together and avoiding monotony (Ferreira et al., 2014), working on improving emotional intimacy (Brotto, Heiman, & Tolman, 2009; Campbell & Rubin, 2012) and communication (Ferreira et al., 2014), and engaging in mindfulness (Brotto & Basson, 2014).

While these strategies can be helpful in maintaining sexual desire, there may be specific strategies that are particularly well suited to cases of sexual desire discrepancy. A recent study found that while couples are generally in sync with their sexual desire (i.e., they ebb and flow at the same time), there may be regular instances of sexual desire discrepancy. The study used spectral and cross-spectral analysis to identify cycles in sexual desire and found that individuals exhibited periodic fluctuations in their desire over the course of a month indicating that there may be regular and predictable fluctuations in desire (Vowels, Mark, Vowels, & Wood, 2018). If desire ebbs and flows naturally, then it is unlikely partners will always be in sync with each other, making desire discrepancy inevitable and potentially problematic for the relationship unless there are strategies employed to mitigate these phases in relationships (Herbenick, Mullinax, & Mark, 2014).

The specific strategies used to mitigate sexual desire discrepancy within long-term relationships have been investigated to a degree in prior research. For example, in a mixed methods study of 179 women in long-term relationships with men, the participants were asked what they do to get their desire back on track when they are out of sync (Herbenick et al., 2014). The authors identified several strategies that the women used to deal with the desire discrepancy including having sex anyway, using toys, being close physically without having sex, or scheduling sex. Most of the participants in the sample also stated that they found the strategies at least somewhat helpful. The study was the first of its kind to try to identify specific strategies that couples may use to help with desire discrepancy. However, the sample consisted of only women and the focus was on getting desire back on track when it is problematic rather than managing naturally occurring and recurring instances of desire discrepancy.

The present paper aimed to build on the study by Herbenick et al. (2014) in several ways by investigating strategies reported by both men and women and by acknowledging that desire discrepancy is a normal part of relationships and thus asking about strategies for when desire discrepancy occurs rather than about ways to get desire back on track. A large-scale study conducted in the U.S. also found that individuals higher in sexual satisfaction and desire reported having more sex, receiving more oral sex, were more likely to incorporate a range of sexual acts, and were more likely to engage in sexual communication (Frederick, Lever, Joseph Gillespie, & Garcia, 2016) and thus the current study also addressed whether using different strategies is associated with sexual and relationship satisfaction and sexual desire. Additionally, due to the different sexual scripts for men and women (Mark & Lasslo, 2018; Murray, 2018), we also examined whether there were differences between men and women in the strategies they used. In order to elicit as many strategies as possible, we used open-ended questions to allow participants to report on any and as many strategies as they wanted. We then used thematic content analysis to code and analyze the qualitative data and did not make any a priori hypotheses on what strategies participants would provide. Our goal was to code individual strategies into groups to enable us to make comparisons between strategies. The approach did not attempt to produce data that are expected to replicate in the future or in other groups, but rather to suggest areas for future study.

The primary purpose of the study was twofold: (1) to identify a variety of strategies that individuals used in long-term relationships to mitigate instances of desire discrepancy using qualitative analysis and (2) to determine whether there were any gender and activity (partner vs. solitary) differences across strategies and whether these strategies differentially predicted sexual and relationship outcomes using quantitative analysis. Specifically, the following four hypotheses were tested: H1: There will be significant gender differences in the strategies men and women use to mitigate instances of desire discrepancy; H2: There will be a significant difference in the frequency of choosing partnered compared to solitary strategies; H3: There will be significant differences in participants’ level of (a) sexual satisfaction, (b) relationship satisfaction, and (c) sexual desire depending on the strategies they use; and H4: Participants who find their strategies helpful will have higher levels of (a) sexual satisfaction, (b) relationship satisfaction, and (c) sexual desire.

## Method

### Participants and Procedure

Prior to commencing data collection, the hypotheses, procedures, and the analysis plan were preregistered on the Open Science Framework (https://osf.io/634us/). Participants were recruited using non-targeted snowball sampling through social media, primarily through sharing a Facebook post through social and professional networks and a tweet on Twitter asking for users to retweet for eligible respondents using hashtags such as #relationshipscience and #sexscience and #relationships. The social media posts linked directly to the link to the study where participants were assessed for eligibility, read the information page, and consented to participate if they were still interested. Participants were also recruited through email listservs and through fliers posted in cafés and libraries around a mid-size city in the U.S. where they were directed to the same eligibility, information, and consent link that initiated participation. The eligibility criteria included being at least 18 years old and currently in a romantic relationship.^1^ As part of a larger project, we aimed to collect between 375 and 500 participants and we finished data collection when the institutional review board approval for data collection finished. Participants completed an online questionnaire and answered demographic questions in addition to measures assessing sexual desire, sexual satisfaction, relationship satisfaction, and open-ended questions about how participants mitigate sexual desire discrepancy on discrepant days in addition to how helpful the strategies were for them. The participants were also asked whether they would like to be entered into a draw to win one of five $50 gift cards as a token of appreciation for participation. All study protocols were approved by the Institutional Review Board of University of Kentucky, and informed consent was obtained from all individual participants included in the study.

For the present study, an a priori power analysis using *G* * Power (Faul, Erdfelder, Lang, & Buchner, 2007) suggested that we would need at least 200 participants, 40 in each of the five groups, in order to detect a medium sized effect (*f* = 0.25) with 80% power with equal sample sizes in each group.^2^ In line with the preregistration, we only included participants who had answered the open-ended questions regarding strategies to dealing with desire discrepancy on a daily basis. A total of 249 participants completed the entire survey, and out of them, a total of 229 (92%) participants had answered the open-ended questions and were thus included in the final analyses, meeting our sample size requirement. The sample consisted of 73 (31.9%) men, 145 (63.3%) women, and 11 (2.8%) genderqueer or genderfluid participants. Furthermore, 213 (93.0%) individuals indicated that their gender identity matched their sex assigned at birth, whereas 12 (5.2%) indicated that their gender identity did not match their sex assigned at birth and four (1.7%) were unsure. The participants were 34.65 years on average (SD = 9.79). The majority of the participants were married or cohabiting (155, 67.7%) followed by in a relationship but living apart (53, 23.2%). Seventeen participants (7.4%) reported being in a consensually non-monogamous relationship. The participants had been in a relationship with their partner for eight years on average (SD = 8.05). The majority of the participants were White (86.5%), well-educated (75.1% had a college degree), non-religious (62.9%), lived in Northern America (76.4%), and 55.5% were heterosexual, 29.7% bisexual, and 5.2% lesbian or gay.

### Measures

The following demographic variables were measured: gender, age, sexual orientation, religion, relationship length, and relationship status. Additionally, participants completed several instruments to assess a number of constructs of interest and open-ended questions, detailed below.

Sexual satisfaction was assessed using the Global Measure of Sexual Satisfaction Scale (GMSEX; Lawrance & Byers, 1992). The GMSEX is a 5-item measure used to assess individual’s sexual satisfaction. The scale is scored on a 7-point semantic differential scale, and higher scores are indicative of greater sexual satisfaction. This scale has shown strong psychometric properties (Mark, Herbenick, Fortenberry, Sanders, & Reece, 2014) ,and in the current sample, the Cronbach’s alpha was .92.

Relationship satisfaction was assessed using the Global Measure of Relationship Satisfaction (GMREL; Lawrance & Byers, 1992). The GMREL measures satisfaction with one’s overall relationship. Similar to the GMSEX, the GMREL is a 5-item scale rated on a 7-point semantic differential and higher scores are indicative of greater relationship satisfaction. This scale has shown strong reliability and validity in previous studies (Lawrance & Byers, 1992), and in the current sample, the Cronbach’s alpha was .94.

Sexual desire was assessed using the Sexual Desire Inventory (SDI; Spector, Carey, & Steinberg, 1996). The SDI is a 14-item, 9-point Likert-scale that assesses an individual’s interest in sexual activity over the past month, and higher scores are indicative of higher sexual desire. The scale can be divided into three subscales: dyadic (partner), dyadic (attractive other), and solitary desire (Moyano, Vallejo-Medina, & Sierra, 2017). For the current study, we preregistered analyses involving the full scale and used dyadic (partner) and solitary scales in the exploratory analyses. The SDI has shown strong evidence of reliability and validity in previous studies (Baumeister, Catanese, & Vohs, 2001), and in the current sample, the Cronbach’s alpha was .90 for all items, .91 for dyadic (partner) desire, and .92 for solitary desire.

The participants were also asked the following questions with open-ended response options: “During times when you feel your desire is higher or lower than your partner’s, what do you do?”, “Does your partner do anything in these cases where one of you has higher or lower desire than the other?”, “Do you and your partner engage in any specific strategies on days when only one of you desires sex?”, and “To what extent do you find these strategies helpful?”

### Analytic Plan

#### Qualitative Coding

A thematic content analysis (Braun & Clarke, 2006) was used in the analysis of the open-ended questions to create categories of similar responses. The initial stage of the analysis was exploratory in nature in which all possible strategies were first compiled into a single list by the first author. She then read the list for an understanding of the themes and categorized them based on similar responses into five overarching themes with subthemes, which were entered into a codebook. The second author read the themes and subthemes for clarity and some of the codes were renamed and some combined. The final codebook consisted of five main themes and 17 subthemes. All participants’ responses were then coded by two independent coders into a main theme and up to three subthemes. If participants mentioned multiple themes, their main theme was based on either their most common answer or the highest group (i.e., the numbers of groups ranged from 1 to 5 and higher numbers indicate greater level of activity). For example, if a participant answered “masturbation” to one of the open-ended options but later indicated that they would masturbate together, the final group would be “engagement in activity together.” If it was not possible to categorize them into one main theme (e.g., participants mentioned a large number of different strategies or their strategy involved being non-monogamous), these participants were not included in the quantitative analyses. Once both coders had coded the responses, they discussed any disagreements until a 100% agreement was reached. We used Cohen’s kappa as a conservative measure of inter-rater reliability (Cohen, 1960; Sim & Wright, 2005). Cohen’s kappa was obtained for each of the main themes and subthemes, and the agreement ranged between .76 and .83 indicating substantial agreement between the coders.

#### Quantitative Analysis

Consistent with the preregistered plan, the five main themes were used to form five groups of individuals who endorsed a particular strategy. Due to the assumption of independence, each participant was only included in one group. For the individual versus partnered strategies analyses, themes “disengagement” and “engagement in activity without a partner” were combined into a single code for “solitary strategies” and “communication,” “engagement in activity with a partner,” and “have sex anyway” were combined into a single code for “partnered strategies.” In the preregistration, our plan was to conduct six one-way ANOVAs to test H3 and H4; however, the assumptions for ANOVA were violated and therefore we proceeded with using the nonparametric Kruskal–Wallis test instead. Thus, instead of using Games Howell post hoc test, we used Dunn’s pairwise tests with Holm–Bonferroni correction as a post hoc test. As specified in the preregistration, three chi-squared tests were used to compare the differences between genders and frequencies of strategies used. In order to control for the experiment-wise error rate often associated with conducting multiple statistical tests, the criteria for statistical significance for the preregistered hypotheses were assessed using the Holm–Bonferroni method (Holm, 1979; Hommel, 1988). In the Holm–Bonferroni method, the *p* values are ordered from the smallest to the largest and the alpha (*p* < .05) is divided by the number of tests (9) that are run. The smallest Holm–Bonferroni corrected alpha level is then compared to the smallest *p* value in the study. Next, the second smallest *p* value is compared to *p* < .05 divided by one less (9–1) than before and this is continued until there are no more significant analyses. The effect sizes are reported as *r* for Kruskal–Wallis and as Cramer’s *V* and log odds for chi-squared analyses. The effect sizes for both r and Cramer’s V conform to 0.1 small, 0.3 medium, and 0.5 large effect size (Cohen, 1988). All analyses were conducted in SPSS 24.0, and effect sizes were calculated using the Excel calculator found on the OSF project page.

Due to non-normal distribution of the outcome variables, we did not impute data using expectation maximization as specified in the preregistration. Instead, we took the mean of the items that were answered if the participants were missing no more than 21% of their data on each scale (Mazza, Enders, & Ruehlman, 2015).^3^ If they missed more than 21%, their responses were not included in the particular analysis.

#### Data Availability Statement

All hypotheses, procedures, and analyses were preregistered, and the data including code to reproduce our analyses are available on Open Science Framework, https://osf.io/634us/. In order to protect the participants’ privacy, the qualitative data were reported separately and participants’ ID and any other identifying data have been removed from the main dataset. Only participants’ main theme and level of helpfulness have been included in the main dataset as these were used in the quantitative analyses.

## Results

### Qualitative Results

*Strategies to Dealing with Desire Discrepancy* The first three questions were used to elicit as many strategies as possible that participants and their partners used by either themselves or together. Participants reported a wide range of strategies they used on days when one partner’s desire was higher or lower than his or her partner’s. Similar strategies were combined together, and these resulted in a total of 17 strategies that were further categorized into main themes, which were used in the quantitative analyses. Each participant could report multiple strategies, but each strategy was counted only once if a participant mentioned the strategy in response to more than one question. A summary of the main themes and subthemes with frequencies and representative quotes can be found in Table 1. The full list of participants’ responses and codes can be found on the OSF project page (https://osf.io/634us/).

**Table 1:** Representative participant quotes for each subtheme from the qualitative responses

Subtheme	n	%	Example quotes
1. Disengagement Do nothing Wait Request for sex but declined Use distractions	23 65 19 13 7	10.7 11.9 3.5 2.4 1.3	“Abstain from activity” “If I want it and she doesn’t, I don’t do anything” “I keep my desire away until he feels like” “If mine is higher and he’s not in the mood then I would wait for him” “I have tried subtle cues to outright asking” “Partner will continually ask for sex. I protest sex or say no to advances” “I might cook a special meal or do some extra chores around the home” “Exercise and rechannel”
2. Communication Communicate Compromise Respect other’s wishes Schedule sex	23 62 11 18 8	10.7 11.4 2.0 3.3 1.5	“Communicate with partner to reassure/assure them of the situation, be supportive to the situation and check into see if there are any problems/concerns/triggers influencing the situation” “Discuss the situation in a way that is mutually understood and positive” “Unless I have a specific reason to do otherwise, I try to motivate myself towards whichever side my partner is on” “Being in a long distance relationship, this rarely happens and desire in person is usually high in both sides. But when it does, we discussed it and compromise, my desire being usually the lower one, he compromises more than I do” “I try to respect that my partner’s desires are not the same as mine, and typically prefer her to initiate any sexual activity” “He’s very understanding of my lack of interest and mostly doesn’t initiate” “Talking about it, making a plan to have a night together with supper, a movie or TV show that we like, and then making sure we both go to bed at a reasonable time with sex being the end goal (that sounds funny, but it works)” “I make an “appointment” with him for a future time”
3. Engagement in activity without partner Masturbate alone Watch porn Read romance novels/erotica	59 122 10 3	27.4 22.3 1.8 0.5	“Masturbate. Happens too often and causing emotional distress and feelings of being undesired” “I usually masturbate a lot to make up for the difference, since I’m nearly always the one who wants sex more often” “I masturbate, watch porn or fantasize” “Watch adult content (porn and non porn)” “Read romance novels” “I masturbate or read erotica”
4. Engagement in activity together Try to trigger desire Different sexual act Spend time non-sexually Other physical closeness	81 54 59 2 30	37.7 9.9 10.8 0.4 5.5	“I am not very good at expressing desire and often take cues from my partner. When I feel desire I try to suggest it by suggesting we take a shower or a similar activity that does not address sex directly” “I wait for my partner to become responsive to my show of affection. I am very passionate and affectionate, she is not at all. I wait until I begin to see some level of intimacy interest and then usual ask if she would like to join me in the bedroom because I am needing that 1-on-1 time. This does not always lead to intercourse but I at least get to be close to her” “Masturbation near each other or engaging in intimate non-sexual activities like showering or massaging” “Partner offers cunnilingus or non-penetrative manipulation of vagina with a toy, or asks for oral/manual manipulation of penis, or masturbates in my presence (with consent), or will wait until alone for masturbation” “I try to spend time with her in a non-sexual scenario” “If someone is disinterested then do something else non-sexual” “Depends on why. We will always kiss, cuddle and stroke, no matter what. Touching each other as often as possible is important to us” “If the desire is there but if either of us is unable to perform (because of tiredness or illness, etc.) then we focus on body connection via sensual touch such as cuddling, massage, or showering with one another”
5. Have sex anyway Have maintenance sex Have sex differently than usual	29 53 10	13.5 9.7 1.8	“Lately it’s been awful, I haven’t desired him, but I have sex with him almost daily because I feel like he emotionally needs the reassurance” “I act as if I want sex, whether I want it or not. At present, it is important to me that sex happens “no matter what,” as I think it is good for the ‘wellbeing’ of the relationship. So, sex has a function right now, and it is to preserve the relationship” “Proceeds with initiation, and judges my continued interest. If it’s relatively still low, we’ll often just have a “wiki wiki” (vanilla, quick-moving, intercourse-driven experience), because that’s fine and pleasurable for us both even if I’m not “I’m the mood,” per se. Usually, though, my own desire “kicks on” and then we play more and extend the experience” “We may be ok with the other partner in more of a submissive/passive role, or be the more submissive/passive role”

The percentages and overall numbers for the main themes were only counted once for each individual. However, each individual may have mentioned more than one subtheme, so the total *N* for the subthemes is greater than the total number of participants in the sample

Masturbation (*n* = 122) was the most common strategy that participants reported to deal with desire discrepancy. Most participants who reported masturbating did not specifically mention whether they masturbated alone or with a partner. However, some were more specific stating either that they masturbated alone, “If one of us is unable to become aroused the other will usually privately masturbate,” or with a partner, “Sometimes he masturbates next to me and I think that’s rather attractive.” One of the participants also expressed desire to masturbate together but stated that her partner was not into it: “Masturbation is the next option. Not mutually masturbation though. I would be totally into that but him not so much.” Some participants also specified that they would watch porn or read romance novels or erotica, which were either used to masturbate privately but also “…to get me in the mood.”

Participants also reported that they would engage in an alternative activity together with most common being a different sexual activity (*n* = 59) followed by trying to trigger desire (*n* = 54) and being close physically without sexual activity (*n* = 30). Different sexual activities included, for example, oral sex, manual stimulation, masturbating together, or using sex toys on each other. One participant said: “When I am in the mood but he isn’t, he would offer to use a vibrator on me or manually stimulate my genital and breasts.” Another participant said: “I would attempt to engage in sex but respect his decision if he is tired and not able to put the effort in. I often then just pleasure him if he’s like that as I enjoy that also.” Many participants also reported that they would try to trigger desire or allow their partner to try to trigger their desire. Some participants felt that the advances were welcome and often successful in stimulating desire. For example, one participant said: “Her desire is almost always higher, and her general strategy tends to be to initiate sex if it’s what she wants. It has a rather high success rate.” However, some participants also reported that triggering desire was not always successful and sometimes triggered negative emotions: “His is generally higher. He tries to get me in the mood, if that fails he can get grumpy.” Other participants chose to be close physically (e.g., cuddling, kissing) without engaging in a sexual activity. For example, one participant said they would “substitute sexual intimacy with other forms of physical intimacy, hugs, cuddling, etc.”

Communication was also important for many participants (*n* = 62). Many of them reported communicating to find out why their level of desire was different: “Talk about it together to work out why.…Talk about when we want sex, because it can be a time of day issue.” Some participants also stated that lack of desire might be due to a misunderstanding, “Talk to them about it. Most of my desire situations have been figured out with talking, and are usually just a misunderstanding of signals,” and others discussed communicating reassurance, “He tries to communicate to me that his lack of desire at that moment is not due to a lack of desire to have sex with me generally.” While communication was generally described as positive, there were also a few participants who described negative communication about desire discrepancy such as complaining, “Complain about my lack of desire or unwanted approaches,” or getting mad, “Kind of ignores that I’m not into it and proceeds; gets mad.”

Despite their lower levels of desire, some participants also reported having maintenance sex (*n* = 53) and reported a variety of reasons for doing so. Some participants viewed this as a positive experience and reported that their desire was responsive: “My desire is often responsive, so I am more likely to agree to sex when I’m not already in the mood.” However, other participants who said they were having sex anyway reported doing so to protect partner’s feelings (“When it’s lower, I try to get excited about having sex anyway to make him happy.”), in order to preserve the relationship (“I act as if I want sex, whether I want it or not. At present, it is important to me that sex happens “no matter what,” as I think it is good for the ‘wellbeing’ of the relationship. So, sex has a function right now, and it is to preserve the relationship.”), or out of obligation (“When it’s lower, I feel an obligation to satisfy his needs.”).

Overall, participants reported a wide range of strategies and many of them also reported multiple strategies to deal with desire discrepancy on days when only one partner desired sex. Notably, many participants (*n* = 65) reported doing nothing when their desire was out of sync. Some participants also explained that they would use different strategies depending on the situation. Participants also indicated that there were times when different strategies were used by them and their partner. However, because we do not have data from both members of the couple and most participants reported similar strategies to both questions, we did not analyze these separately.

*Helpfulness of Strategies* The final open-ended question was related to how helpful participants found the strategies they used. We were able to categorize 149 responses. The rest of the participants either left the question blank or indicated that the question did not apply to them. Half of the participants indicated that they found the strategies used very helpful (*n* = 73, 49.0%). These participants talked about how they felt closer as a result: “I think for the most part my husband and I communicate pretty well, and are pretty good at getting our needs met. I feel very satisfied with the sexual part of our relationship. It’s always been one of the most consistently positive parts of my life.” Some participants also talked about the desire to please each other even when own desire may be lower: “I think it makes both of us happy to please the other person sexually even if one of us doesn’t feel strong desire in a particular moment.” Those who thought their strategies were very helpful included those who communicated (57.1%), engaged in another activity together (53.8%), or had sex anyway (57.9%). Less than half the participants who engaged in activity alone (45.7%) and only a minority of the participants who said they disengaged (9.1%) found their strategies very helpful.

A total of 51 participants found their strategies at least somewhat helpful (34.2%). One participant said “It’s a bit of a toss of a coin but we both have our variations so I guess we just got used to them” and another participant said “Meh, sometimes you win some and sometimes you lose some. Masturbation gets me off but it sometimes makes me feel more disconnected since he isn’t willing to please me.” The statements suggest that the strategies may work sometimes or deal with an immediate problem but do not feel completely satisfying in the long-term. At least one-third of the participants who said they communicated (42.9%), engaged in activity alone (34.3%), engaged in another activity together (33.8%), or had sex anyway (36.8%) found their strategies at least somewhat helpful. In contrast, only a minority of participants who reported disengaging (18.2%) found their strategy at least somewhat helpful.

Finally, 25 participants indicated that their strategies were not helpful (16.8%). Some of the participants who were unhappy with their strategies wished they were able to engage in activity together. For example, one of the participants said “Not at all - my desire is very partner specific so masturbation doesn’t give me what I want.” Other participants talked about how they experienced negative feelings as a result of the strategies not being helpful: “Isolating not having a strategy or being able to communicate the importance to either side.” Most of the participants who reported disengaging stated that their strategies were not helpful (72.7%) compared to only a minority of the participants who endorsed at least some form of activity: engaging in activity alone (20.0%), engaging in another activity with a partner (12.3%), and having sex anyway (5.3%). None of the participants who reported communicating about their desire discrepancy reported their strategy unhelpful.

### Confirmatory Quantitative Analyses

The results of a chi-squared test indicated a significant difference between men and women (*χ*^2^[4] = 14.99, *p* = .005, Cramer’s *V* = .27) in the strategies they chose, providing support for H1. We did not specify any post hoc comparisons in the preregistration, and therefore the comparisons are exploratory. We used the adjusted residual scores to compute a chi-squared score and corresponding *p* values, which were adjusted for multiple comparisons using the Holm–Bonferroni correction. None of the post hoc comparisons were significant. Next, we explored the possibility that men and women differed in solitary versus partnered strategies and indicated that women were 2.55 times more likely to report partnered strategies compared to men (*χ*^2^ (1) = 9.49, *p* = .002, Cramer’s *V* = .22).

In order to address H2, we conducted two chi-squared tests: one to compare the frequency of choosing solitary strategies compared to partnered strategies and one to compare the frequency of choosing each strategy. The results provided support for the hypothesis and indicated that participants were 1.63 times more likely to endorse partnered strategies (*n* = 134) compared to solitary strategies (*n* = 82), *χ*^2^(1) = 12.52, *p* < .001. Further, participants were 1.37 times more likely than expected to report engaging in activity alone (*n* = 59) and 1.88 times more likely than expected to engage in another activity together (*n* = 81). In contrast, they were 1.87 times less likely than expected to report doing nothing (*n* = 23), 1.87 times less likely than expected to report communicating with partner (*n* = 23), and 1.48 times less likely than expected to report having sex anyway (*n* = 29), *χ*^2^(4) = 62.70, *p* < .001.

A series of Kruskal–Wallis H tests were used to answer H3. There were significant differences between strategies in the participants’ level of sexual satisfaction: *H*(4, *N* = 215) = 18.65, *p* = .001, *r* = .29. Dunn’s pairwise tests with Holm–Bonferroni correction (ten comparisons) were used to compare strategies and found that doing nothing was significantly worse for sexual satisfaction than engaging in activity alone (*p* = .006), communicating (*p* = .001), engaging in activity together (*p* < .001), or having sex anyway (*p* = .001). None of the other comparisons were significant after correcting for the familywise error rate. The results also indicated that there were significant differences between strategies in the participants’ level of relationship satisfaction: *H*(4, *N* = 215) = 17.85, *p* = .001, *r* = .28. Dunn’s pairwise tests with Holm–Bonferroni correction (ten comparisons) were used to compare strategies and indicated that doing nothing was significantly worse for relationship satisfaction than communicating (*p* < .001) or engaging in activity together (*p* = .002). None of the other comparisons were significant after correcting for the familywise error rate. After applying the Holm–Bonferroni correction for the main tests, there were no significant differences in levels of sexual desire across groups: *H*(4, *N* = 215) = 10.12, *p* = .038, *r* = .21. Thus, the results provided support for the H3a and H3b but not H3c.

Finally, we conducted a series of Kruskal–Wallis tests to assess whether individuals who found their strategies very helpful, somewhat helpful, and not at all helpful differed in their levels of sexual and relationship satisfaction and sexual desire. We predicted that participants who found their strategies more helpful would have higher levels of sexual and relationship satisfaction and desire. The prediction was directional, and therefore the *p* values are adjusted for one-tailed alpha level. There were significant differences with large effect sizes between groups in their level of sexual satisfaction: *H*(2, *N* = 149) = 43.63, *p* < .001, *r* = .48. Dunn’s pairwise tests with Holm–Bonferroni correction were conducted to determine which groups significantly differed from one another. People who did not find their strategies helpful had lower levels of sexual satisfaction compared to individuals who found their strategies somewhat helpful (*p* = .006) and very helpful (*p* < .001). Individuals who found their strategies very helpful also had higher sexual satisfaction compared to those who only found them somewhat helpful (*p* < .001). Groups also differed in their level of relationship satisfaction *H*(2, *N* = 149) = 21.43, *p* < .001, *r* = .36. Dunn’s pairwise tests with Holm–Bonferroni correction indicated that people who did not find their strategies helpful had lower levels of relationship satisfaction compared to individuals who found their strategies somewhat helpful (*p* = .002) and very helpful (*p* < .001). Individuals who found their strategies very helpful also had higher relationship satisfaction compared to those who only found them somewhat helpful (*p* = .025). Finally, there were no significant differences between groups in their level of sexual desire *H*(2, *N* = 149) = 2.90, *p* = .118, *r* = .14. Therefore, the findings provided support for H4a and H4b but not for H4c.

### Exploratory Quantitative Analyses

Although not specified in the preregistration, we also conducted Kruskal–Wallis H tests to compare solitary and partnered strategies. Because these hypotheses were not specified a priori, they should be interpreted as exploratory and were not part of the Holm–Bonferroni corrected tests. Participants who engaged in partnered strategies were significantly more sexually satisfied compared to participants who engaged in solitary strategies: *H*(1, *N* = 216) = 11.53, *p* = .001, *r* = .23. The participants in the partnered strategies group were also significantly higher in their levels of relationship satisfaction: *H*(1, *N* = 216) = 5.94, *p* = .015, *r* = .16. Finally, there were no significant differences between groups in their level of sexual desire: *H*(1, *N* = 216) = 2.43, *p* = .12, *r* = .11.

Because the chi-squared analyses indicated significant differences between men and women in the strategies they chose, we decided to also examine whether the strategies associated with sexual and relationship satisfaction and sexual desire differed for men and for women. First, men and women did not significantly differ in their level of sexual (*M* = 5.11, SD = 1.64 for men and *M* = 5.57, SD = 1.71 for women; *p* = .059) or relationship (*M* = 5.76, SD = 1.29 for men and *M* = 6.06, SD = 1.21 for women; *p* = .096) satisfaction, but men reported significantly higher levels of sexual desire compared to women (*M* = 5.08, SD = 1.96 for men and *M* = 44.31, SD = 1.95 for women; *p* = .007). The results for the Kruskal–Wallis H tests comparing all five groups indicated that men significantly differed only in their sexual satisfaction based on the strategies they used *H*(4, *N* = 66) = 11.52, *p* = .021, *r* = .40. The post hoc tests with Holm–Bonferroni correction indicated that there was only a significant difference between doing nothing and communication (*p* = .002) with participants who reported communicating with their partner showing higher levels of sexual satisfaction. For women, there were significant differences in both sexual, *H*(4, *N* = 139) = 9.86, *p* = .043, *r* = .26, and relationship satisfaction, *H*(4, *N* = 139) = 11.35, *p* = .023, *r* = .28. The post hoc tests indicated a significant difference between doing nothing and communicating (*p* = .002) in that participants who reported communicating with their partner experienced higher levels of relationship satisfaction compared to the women who reported doing nothing. None of the other comparisons were significant after controlling for the familywise error rate. We also compared solitary and partnered strategies for men and women separately. There were no significant findings for men, but women who reported using partnered strategies were more sexually satisfied compared to women who reported using solitary strategies, *H*(1, *N* = 140) = 7.05, *p* = .008, *r* = .22.

Furthermore, men differed significantly in their level of relationship satisfaction based on how helpful they found the strategies, *H*(2, *N* = 43) = 16.68, *p* < .001, *r* = .54. More specifically, men who found their strategies very helpful were significantly more satisfied in their relationship compared to men who found their strategies only somewhat helpful (*p* = .017) or not at all helpful (*p* < .001). Men who found their strategies somewhat helpful were also more satisfied in their relationships compared to men who did not find the strategies helpful (*p* = .012). Similarly, men also differed in their level of sexual satisfaction based on the level of helpfulness, *H*(2, *N* = 43) = 23.44, *p* < .001, *r* = .61. Specifically, men who found their strategies very helpful were significantly more sexually satisfied compared to men who found their strategies only somewhat helpful (*p* < .001) or not at all helpful (*p* < .001). Men who found their strategies somewhat helpful were also more sexually satisfied compared to those who did not find their strategies helpful (*p* = .029). For women, there were only significant differences in their level of sexual satisfaction, *H*(2, *N* = 99) = 15.30, *p* < .001, *r* = .37. Women who found their strategies very helpful were significantly more sexually satisfied compared to women who found their strategies only somewhat helpful (*p* = .007) or not at all helpful (*p* = .001). None of the analyses were significant for sexual desire.

We used the full-scale score of the SDI for the confirmatory analyses but were also interested in whether there would be significant group differences in dyadic sexual desire and solitary sexual desire. We tested the differences between the five main groups as well as solitary and partnered strategies, but there were no significant differences across groups. We also ran additional analyses to see whether there were significant differences in the strategies chosen based on sexual orientation. However, we only had large enough sample sizes within the bisexual and heterosexual groups and were therefore only able to compare these two groups. There were no significant differences between straight and bisexual individuals in either the main themes or solitary and partnered strategies. We also grouped participants into religious and non-religious individuals to allow us to compare these groups. It would not have been possible to compare all religions due to relatively small sample sizes in each group. There were no significant differences between participants who identified with a specific religion compared to those who identified as atheist or as non-religious in either the main strategies or solitary versus partnered strategies.

## Discussion

We recognize that ebbs and flows are a natural part of the experience of sexual desire (Acevedo & Aron, 2009; Herbenick et al., 2014; Vowels et al., 2018). Within relationships, sexual desire discrepancy is an inevitable experience and can be difficult for couples to navigate successfully. Thus, the purpose of the present study was to identify strategies that people use in their daily lives to deal with desire discrepancy as it arises in relationships and to compare the effectiveness of these strategies with a view of helping researchers, clinicians, and educators to provide evidence-based strategies that work for couples struggling with desire discrepancy.

The qualitative analyses revealed a variety of strategies used to mitigate sexual desire discrepancy in relationships. The most common strategies indicated were masturbation, engaging in a different sexual activity, communication, having sex regardless of the lower level of desire, and doing nothing. Note that most participants defined having sex as vaginal penetration and classified other forms of sexual activity (e.g., oral sex, manual stimulation) as not having sex. Many of these strategies were similar to the ones identified by Herbenick et al. (2014) suggesting they can be used as a way to deal with desire discrepancy when it occurs but also as a way of getting back on track. The relative frequency of certain strategies differed, however, potentially reflecting our study design, which included both men and women and asked specifically about maintenance within the relationship. Masturbation, for example, may be more of a way to deal with immediate desire discrepancy on a day-by-day basis rather than something someone might cite as important for getting back on track with their partner related to sexual desire.

Furthermore, a majority of the participants in our study who said they did nothing found their strategies unhelpful, whereas at least half the people who engaged in a partnered activity whether that was communication, having sex, or engaging in an alternative activity together found their strategies very helpful. The split was more even in the group of participants who reported masturbating. In addition to the strategies discussed in the results-section, we also had a subsample of polyamorous couples who often reported having sex with another partner if their primary partner was not interested in having sex. All the participants (available *n* = 10) who reported being in a consensually non-monogamous relationship found their strategies at least somewhat helpful. For example, one participant stated, “I’ve found polyamory to be very helpful at putting less pressure on the sexual side of a relationship,” and another participant said, “Polyamory helps with these balance issues. We each get what we need from the others and respect the ups and downs of desire.” However, we did not include consensual non-monogamy as a strategy in the present study as this would not generalize into other couples who are not consensually non-monogamous.

The analyses comparing frequencies of specific strategies indicated that participants were more likely to report engaging in a different sexual activity or masturbating and less likely to report doing nothing, communicating, or having sex anyway. People were also more likely to endorse partnered compared to solitary activities. Furthermore, men and women may differ in the strategies they use and exploratory analyses indicated that women were more likely to report engaging in partnered activities compared to men who were more likely to report solitary activities. Prior research has shown that women’s sexual desire may decrease as relationship length increases (Ainsworth & Baumeister, 2012; Klusmann, 2002; Murray & Milhausen, 2012a), which may contribute to the differences in strategies. Furthermore, it has been more acceptable societally for men to engage in masturbation compared to women (Fahs & Frank, 2014; Kaestle & Allen, 2011), which may also explain why women are less likely to report solitary activities.

The results also indicated that people who did nothing to combat desire discrepancy and disengaged from their partner had lower levels of relationship and sexual satisfaction compared to those who addressed the desire discrepancy. Participants who engaged in communication or an alternative activity with a partner compared to disengaging from their partner also reported higher levels of relationship satisfaction and participants who engaged in any activity had higher levels of sexual satisfaction. Further, individuals who found their strategies very helpful had highest levels of both relationship and sexual satisfaction. These findings are consistent with previous research suggesting that engaging in more sex, different sexual activities, and sexual communication are linked with higher levels of sexual satisfaction (Frederick et al., 2016). Due to the significant differences in strategies reported between men and women, we also repeated the analyses for men and women separately. However, because of the exploratory nature of these analyses and a small sample, caution should be used when interpreting these findings and future research should further investigate the potential gender differences. None of the findings for sexual desire were significant. This suggests that the differences in sexual and relationship satisfaction were not due to different levels of sexual desire across groups but rather the way participants dealt with desire discrepancy. These results show that addressing and dealing with desire discrepancy is important for relationships and some strategies may be more successful than others.

Our study adds to the current literature by specifically identifying strategies that men and women use to deal with desire discrepancy in relationships. We also demonstrated that the types of strategies and helpfulness of these strategies matter for sexual and relationship satisfaction. The results were not only significant but the effect sizes were also notable, suggesting that these findings also have practical significance. Additionally, we addressed the questions using both qualitative and quantitative methods, which allowed us to quantify our findings but to also provide nuanced information about participants’ lived experiences. Our findings also have implications for clinicians and educators who work with desire discrepancy and highlight the importance of discussing management of desire discrepancy with couples and providing evidence-based suggestions. The findings also add to our theoretical understanding of how to manage desire discrepancy in couple relationships.

### Limitations and Future Research

Despite the many strengths of the study, the study also had several limitations. First, there is always some level of bias when analyzing qualitative data and placing people into one group was not always clear cut. For example, previous literature has shown that people who have sex for approach-related goals have higher levels of satisfaction compared to those who have sex for avoidance-related reasons (Impett, Strachman, Finkel, & Gable, 2008). Many participants did not list the reason why they engaged in a particular strategy and thus we were unable to separate these people into different groups and everyone who reported having sex were grouped together. This was particularly notable in the partner-focused strategies, where something like attempting to trigger desire for one’s partner is distinct from engaging in a different sexual activity or in something that was physically intimate to meet a partner’s needs. Similarly, many participants did not report whether they masturbated alone or with a partner and we assumed that unless people reported masturbating with their partner, they were masturbating alone. Additionally, our partnered strategies included reasons that were specific to meeting a partner’s needs through respecting their partner. Some of these decisions were not preregistered as we were not aware of the need until after these data were collected. Future research should address these limitations by listing potential strategies and letting participants choose ones that best fit their experience to be able to better categorize these experiences. Future research could also examine the ways in which participants are interpreting the questions. Second, our results rely on cross-sectional data and therefore we cannot make any causal claims about the direction of our findings. For example, it may be possible that individuals with higher sexual and relationship satisfaction are more likely to want to engage in a partnered activity. Third, we only collected data from individuals and were thus unable to address the impact of the strategies for both partners in the relationship. Future research should collect longitudinal data from couples to address these limitations. For example, a daily diary study of couples reporting on strategies they use on days when only one partner desires sex would provide a stronger design. We hope that our findings can serve as a basis for future studies of this kind. Fourth, even though our sample was diverse in terms of their age, gender, sexual orientation, and relationship status, most of the participants were White, well-educated, and non-religious. It would be ideal for future research to replicate our findings in more ethically and socioeconomically diverse samples. Fifth, we asked open-ended questions to explore what participants would spontaneously respond to question about strategies they used when they experienced desire discrepancy, but recognition memory may be more accurate. Therefore, future studies concerned with the accuracy or counts of the report might choose to offer response options as well as an open format.

### Conclusion

The findings of the present study add to the literature on maintaining sexual desire in long-term relationships by focusing specifically on strategies to dealing with desire discrepancy when it occurs. Our findings indicated that people use a variety of strategies to deal with desire discrepancy, some of which are more helpful than others. We also found that choosing a particular strategy can have implications for sexual and relationship satisfaction and choosing partnered activities can be more beneficial than choosing a solitary activity. Our findings provide practical information to researchers, clinicians, and educators working with couples and highlight the importance of communication about desire in relationships and using effective strategies to help during times when desire levels are discrepant for partners. Future research is needed to address some of the limitations of the present study and our hope is that our study can act as a catalyst for this research.

## Footnotes

1 We did not specify a minimum relationship length but reran the analyses including only participants who had been in a relationship for at least 6 months duration (*n* = 219). Excluding participants with shorter relationship length (10 participants) did not change the results.

2 We did not know how many groups there would be a priori and also ran a power analysis for four groups which would have required 180 participants divided into four groups.

3 A cutoff of 20% is seen as standard in the literature (Mazza et al., 2015). Because the sexual desire scale comprises 14 items, we retained participants who had missing data on three items or less resulting in a cutoff of 21%.
